# Association Between the Number of Intratympanic Steroid Injections and Hearing Recovery in Sudden Sensorineural Hearing Loss

**DOI:** 10.3389/fneur.2021.798569

**Published:** 2021-12-14

**Authors:** Yixu Wang, Ge Gao, Le Wang, Xin Ma, Lisheng Yu, Fanglei Ye

**Affiliations:** ^1^Department of Otorhinolaryngology, Head and Neck Surgery, People's Hospital, Peking University, Beijing, China; ^2^Department of Radiology, First Hospital, Peking University, Beijing, China; ^3^Department of Otorhinolaryngology, Head and Neck Surgery, First Affiliated Hospital of Zheng Zhou University, Zhengzhou, China

**Keywords:** intratympanic steroid, hearing recovery, sudden sensorineural hearing loss, intratympanic numbers, treatment

## Abstract

The frequency of intratympanic (IT) steroid injection varies from once daily to once weekly or less among studies and does not reach a uniform standard. This study investigated the potential association between the number of IT steroid injections and hearing recovery to determine the optimal number in sudden sensorineural hearing loss (SSNHL) patients. A retrospective study involving 233 SSNHL patients receiving IT steroids plus batroxobin within 7 days of onset was performed. Patients were followed up for 3 months. More than 15 dB of HL improvement in the pretreatment pure tone average (PTA) was defined as effective. The effective group had a higher IT injection numbers than the ineffective group (≥ 6 times: 84.6 vs. 61.1, *p* < 0.001). Regardless of the unadjusted model or adjusted model, patients who received more frequent IT steroid injections seemed more likely to recover hearing (unadjusted model, OR, 95% CI: 1.25, 1.06–1.48; *p* = 0.007; adjusted model, OR, 95% CI: 1.21, 1.01–1.45; *p* = 0.044). Six IT injections had the highest rate of hearing recovery (79.1%). In conclusion, IT injection number was an independent factor that was positively associated with hearing recovery, and the optimal number of IT steroid injections was 6. Batroxobin plus higher number of IT steroid injections showed more effective for treating SSNHL.

## Introduction

Sudden sensorineural hearing loss (SSNHL) is characterized by hearing loss >30 dB occurring at least 3 consecutive frequencies within 3 days (1). SSNHL affects 5–27 per 100,000 people annually, with approximately 66,000 new cases per year in the United States (2–4). Many treatment options, such as steroids, hyperbaric oxygen therapy, antiviral agents, rheologic agents, and other medications, have been recommended for SSNHL (5). Among these, steroids are commonly confirmed to be effective and are recommended by the guidelines for SSNHL set by the AAO-HNS in 2019 (1, 6). Generally, steroids can be given via systemic or intratympanic (IT) therapy. IT steroids have recently become popular because of their effectiveness, and they can avoid many of the side effects caused by systemic steroid injection, such as acne, increased appetite, hyperglycemia, hypertension, and gastric irritation (7). Together with many other studies, we previously found that IT injection could result in higher drug concentrations in the perilymph and cochlea than systemic injection in animals and suggested that IT injection may be more appropriate for SSNHL (8–10).

Currently, the frequency of IT steroid injection varies from once daily to once weekly or less among studies and does not reach a uniform standard (7, 11, 12). A higher frequency may not be more effective due to the limited glucocorticoid receptors (GRs) that steroids must bind to take an effect. Additionally, frequent IT injections could be expensive and cause many adverse effects, including pain, transient dizziness, infection, and persistent tympanic membrane perforation (13). It is appropriate to determine the optimal number of IT injections for reducing costs and side effects and having an optimal effect. This study extends our previous work to investigate the potential association between IT injection number and hearing recovery to determine the optimal number of IT injection.

## Materials and Methods

### Study Design

This retrospective study was approved by the regional ethical standards committee of the First Affiliated Hospital of Zhengzhou University. According to Siegel's criteria and guidelines for sudden hearing loss issued by the Chinese Society of Otorhinolaryngology Head and Neck Surgery in 2015, more than 15 dB of HL improvement in the pretreatment PTA was defined as effective; lower levels of improvement were defined as ineffective (14, 15). According to the therapeutic effect of IT steroids, patients were divided into effective and ineffective groups. This study consisted of two parts. First, we compared the clinical characteristics, hearing characteristics, and IT times between the effective and ineffective groups to determine the potential factors associated with hearing recovery. Second, based on the results of Part 1, we used logistic regression analysis in unadjusted and adjusted models to investigate the association between IT times and hearing recovery and determine the optimal IT times for SSNHL patients.

### Patients

We reviewed the clinical data of new, consecutive SSNHL patients visiting the otolaryngology head-neck surgery department inpatient at the First Affiliated Hospital of Zhengzhou University between January 2017 and December 2020. All patients had an extensive history taken and underwent a series of examinations, including physical examination, neurotological examination, imaging, audiology, and vestibular testing. The studied patients received comprehensive treatment, including treatment with IT steroids and intravenous batroxobin (10 U batroxobin for the first treatment, which was then reduced to 5 U batroxobin, once every other day, for a total of 3–5 times according to the fibrinogen level), based on the Chinese guidelines for SSNHL (15). Batroxobin was a thrombin-like serine protease from the venom of the Bothrops atrox and Bothrops moojeni snake species (16). Batroxobin could reduce the fibrinogen level in blood and used for many ischemic disorders (17, 18). IT steroids were injected once a day within the first four days, followed by every other day for the remaining days. IT steroid injection was stopped when (1) patients were unwilling to continue IT steroids, (1) patients could not bear the pain or transient dizziness, (3) inflammation in the external auditory canal or tympanic membrane occurred, or (4) vasovagal or syncopal episodes occurred during injection. The number of IT steroid numbers would be determined by the above factors. The patients received 0.5-ml dexamethasone of 10 mg/mL over two weeks, with a dose given through the tympanic membrane into the middle ear by an otolaryngologist using an operating microscope. Anesthesia was achieved with topical phenol. Patients were positioned supine with the affected ear slightly up and remained in this position for 30 min after the injection. They were instructed to keep water out of their treated ear for the duration of treatment. They were all instructed to undergo an audiological examination after 7 days of treatment, 14 days of treatment, and 3 months of follow-up. The diagnostic criteria for SSNHL included a sensorineural hearing loss of more than 30 dB, occurring at least three contiguous frequencies within 72 h. Additional inclusion criteria were as follows: (1) age between 20 and 65 years; (2) time prior to treatment was not more than a week; and (3) their first treatment was at our hospital. The major exclusion criteria included a previous history of SSNHL in either ear, history of fluctuating hearing, Ménière's disease, history of ear surgery, history of otosclerosis, congenital hearing loss, conductive hearing loss, physical trauma or barotrauma to the ear, history of genetic hearing loss with strong family history, or craniofacial or temporal bone malformations.

### Pure Tone Audiogram

Hearing tests were performed by air- and bone-conducted pure tone audiometry and speech audiometry at screening, after 1 and 2 weeks of treatment, and at 3 months of follow-up. According to the guideline for SSNHL ruled by Chinese Society of Otorhinolaryngology Head and Neck Surgery in 2015 (15), the pure tone audiograms were categorized into four types: high- or low-frequency hearing loss, flat-type hearing loss, or profound hearing loss based on their shapes. Low-frequency hearing loss was defined as having more than 30 dB of average hearing loss at ≤ 1 kHz (at least at 0.25–0.5 kHz). High-frequency hearing loss was defined as having more than 30 dB of average hearing loss at≥2 kHz (at least at 4–8 kHz). In the flat-type hearing loss type, the average hearing thresholds of 0.25, 0.5, 1, 2, 4, and 8 kHz frequencies were <80 dB. In the profound hearing loss type, the average hearing thresholds of 0.25, 0.5, 1, 2, 4, and 8 kHz frequencies were more than 81 dB. Other types were not included in this study, because they were rare in China. The PTA was calculated with hearing thresholds of 0.5, 1, 2, and 4 kHz.

### Statistical Analysis

SPSS software (version 21.0, SPSS) was used for statistical analyses. For categorical data, frequencies and percentages were calculated to describe the distributions of subgroups among patients according to sex, tinnitus, aural fullness, vertigo, location of damaged ear, history of diabetes or hypertension, audiogram shapes, number of IT injections and prognosis effect. Categorical data were compared with the chi-squared test or Fisher's exact test if appropriate. Means (±SD) were used to summarize the average levels of quantitative data, such as age, initial treatment time, PTA and number of batroxobin injections. The Shapiro–Wilk test was used to examine whether the measured values could be approximated by a normal distribution. Quantitative data that were distributed normally were compared by a two-sample *t*-test; those that were distributed non-normally were compared by the Wilcoxon signed-rank test. A binary logistic regression analysis in unadjusted and adjusted models was conducted to determine the association between the number of IT steroid injections and hearing recovery. ORs and 95% confidence intervals were calculated according to model-variable coefficients and standard errors, respectively. All *p* < 0.05 indicated statistical significance.

## Results

### Baseline Characteristics of SSNHL Patients

During the study period, 763 SSNHL patients were included. Among these patients, 233 patients met the inclusion criteria, and 530 were excluded according to the exclusion criteria. The mean (SD) age of the patients was 44.15 ± 11.71 years, and 48.9% were women. Vertigo, tinnitus and aural fullness were present in 48.1%, 84.5% of patients and 65.7% of patients, respectively. The mean hearing threshold of the affected ear was 72.30 ± 31.85 dB. After analyzing the audiograms, 14.2% indicated low-frequency hearing loss; 16.7%, high-frequency hearing loss; 32.2%, flat-type hearing loss; and 36.9%, profound hearing loss. The mean number of IT steroid injections was 6.69 ± 1.64 injections. After treatment, 61.4% of treatments were found to be effective (Table 1).

**Table 1:** Baseline. Hearing and prognosis characteristics of SSNHL patients.

**Characteristics**	**All studied patients (*n* = 233)**
Age, mean, y	44.15 ± 11.71
Women, No. (%)	114 (48.9)
Left affected ear	124 (53.2)
Pure tone average, mean, dB	
Affected ear	72.30 ± 31.85
Shapes of audiogram, No. (%)	
Low-tone hearing loss	33 (14.2)
High-tone hearing loss	39 (16.7)
Flat type	75 (32.2)
Profound loss	86 (36.9)
Other aural symptom, No. (%)	
Vertigo	112 (48.1)
Aural fullness	153 (65.7)
Tinnitus	197 (84.5)
Hypertension, No. (%)	33 (14.2)
Diabetes, No. (%)	20 (8.6)
Initial treatment time, mean, day	4.63 ± 2.00
Number of IT steroid injections, No. (%)	
<6 times	57 (24.5)
≥6 times	176 (75.5)
Number of batroxobin, mean, times	4.66 ± 0.94
Adverse event, No. (%)	
Pain	96 (41.2)
Dizziness/vertigo	92 (39.5)
Ear infection	0 (0.00)
Tympanic membrane perforation	10 (4.3)
Prognosis, No. (%)	
Recovery	143 (61.4)

### Comparison of Hearing Characteristics and Number of IT Injections Between the Effective and Ineffective Groups

Compared to the ineffective group, the effective group was younger (40.81 ± 11.74 vs. 49.46 ± 9.54, *P* < 0.001). The symptom of vertigo differed significantly between the effective and ineffective groups (41.3 vs. 58.9%, *P* = 0.011). The effective group had a shorter initial treatment time than the ineffective group (4.36 ± 1.94 vs. 5.06 ± 2.03, *P* = 0.010). Audiogram shapes also differed significantly between the effective and ineffective groups (*P* = 0.001). Compared to the ineffective group, the effective group had higher IT injection numbers (≥6 times: 84.6 vs. 61.1%, *p* < 0.001). However, sex, location of affected ear, PTA before treatment, number of batroxobin injection, symptoms of aural fullness and tinnitus, hypertension and diabetes did not differ significantly between the effective and ineffective groups (Table 2).

**Table 2:** Comparison of prognosis between effective group and ineffective group.

**Characteristics**	**Effective**	**Ineffective**	***P*-value**
	**group**	**group**	
	**(*n* = 143)**	**(*n* = 90)**	
Age, mean, y	40.81 ± 11.74	49.46 ± 9.54	<0.01
Women, No. (%)	79 (55.2)	45 (50.0)	0.500
Left affected ear	53 (58.9)	71 (49.7)	0.169
Pure tone average, mean, dB			
Affected ear	69.84 ± 32.08	76.21 ± 31.26	0.138
Shapes of audiogram, No. (%)			0.001
Low-tone hearing loss	31 (21.7)	2 (2.2)	
High-tone hearing loss	21 (14.7)	18 (20.0)	
Flat type	44 (30.8)	31 (34.4)	
Profound loss	47 (32.9)	39 (43.3)	
Other aural symptom, No. (%)			
Vertigo	59 (41.3)	53 (58.9)	0.011
Aural fullness	90 (62.9)	63 (70.0)	0.798
Tinnitus	124 (86.7)	73 (81.1)	0.322
Hypertension, No. (%)	19 (13.2)	14 (15.5)	0.239
Diabetes, No. (%)	10 (7.0)	10 (11.1)	0.338
Initial treatment time, mean, day	4.36 ± 1.94	5.06 ± 2.03	0.010
Number of IT steroid injections, No. (%)			<0.001
<6 times	22 (15.4)	35 (38.9)	
≥6 times	121 (84.6)	55 (61.1)	
Number of batroxobin, mean, times	4.63 ± 0.92	4.71 ± 0.99	0.521

*Quantitative data, such as age, initial treatment time, PTA and number of IT injections was compared by a two-sample t-test or Wilcoxon signed-rank test. Categorical data, such as sex, tinnitus, aural fullness, vertigo, location of damaged ear, history of diabetes or hypertension and audiogram shapes was compared by chi-squared test or Fisher's exact test*.

### Association Between Number of IT Injections and Hearing Recovery

After comparing the effective and ineffective groups, age, symptoms of vertigo, initial treatment time, audiogram shapes and number of IT injections seemed to be potential factors associated with hearing recovery. We used a logistic regression analysis in unadjusted and adjusted models to explore the association between the number of IT injections and hearing recovery. The adjusted model was adjusted for potential factors such as age, symptoms of vertigo, initial treatment time, and audiogram shapes. Regardless of the unadjusted model or adjusted model, patients who received more frequent IT steroid injections seemed more likely to recover hearing (unadjusted model, OR, 95% CI: 1.25, 1.06–1.48; *p* = 0.007; adjusted model, OR, 95% CI: 1.21, 1.01–1.45; *p* = 0.044; Table 3). After further analysis of the effectiveness of the number of IT injections, we found that 6 injections could cause the highest rates of hearing recovery. When more than 6 IT injections were administered, the rate of hearing recovery no longer increased (Table 4).

**Table 3:** Potential factors associated with hearing recovery.

	**Unadjusted model**		**Adjusted model**	
**Variable**	**OR (95% CI)**	***P*-value**	**OR (95% CI)**	***P*-value**
Age, mean,y	0.93 (0.90,0.96)	<0.001		
Vertigo, No. (%)	0.49 (0.29,0.84)	0.009		
Initial treatment time	0.84 (0.73,0.96)	0.011		
Audiogram shapes				
Low-tone hearing loss	12.86 (2.89, 57.16)	0.001		
High-tone hearing loss	0.97 (0.45, 2.07)	0.968		
Flat type	1.18 (0.63,2.20)	0.608		
Profound loss	Ref (1.00)			
IT times	1.25 (1.06,1.48)	0.007	1.21 (1.01, 1.45)	0.044

*Adjusted model: adjusted for age, syptom of vertigo, audiogram shapes, and initial treatment time*.

**Table 4:** Comparision of baseline. Hearing and prognosis characteristics in SSNHL patients according to IT injection number.

**Characteristics**	**4 Injections**	**5 Injections**	**6 Injections**	**7 Injections**	**8 Injections**	**9 Injections**	***P*-value**
Age, mean, y	48.49 ± 9.57	44.83 ± 9.17	43.77 ± 11.11	43.74 ± 13.46	42.74 ± 12.50	42.20 ± 10.70	0.202
Women, No. (%)	19 (48.7)	8 (44.4)	25 (58.1)	21 (55.3)	28 (42.4)	13 (44.8)	0.619
Left affected ear	20 (51.3)	10 (55.6)	26 (60.5)	16 (42.1)	36 (54.5)	16 (55.2)	0.703
Pure tone average, mean, dB							
Affected ear	68.81 ± 33.82	58.26 ± 39.82	72.76 ± 31.23	75.36 ± 31.76	78.64 ± 29.51	65.26 ± 27.92	0.149
Shapes of audiogram, No. (%)							0.888
Low-tone hearing loss	6 (15.4)	2 (11.1)	8 (18.6)	5 (13.2)	8 (12.1)	4 (13.8)	
High-tone hearing loss	5 (12.8)	5 (27.8)	6 (14.0)	5 (13.2)	10 (15.2)	8 (27.6)	
Flat type	13 (33.3)	5 (27.8)	14 (32.6)	12 (31.6)	20 (30.3)	11 (37.9)	
Profound loss	15 (38.5)	6 (33.3)	15 (34.9)	16 (42.1)	28 (42.4)	6 (20.7)	
Other aural symptom, No. (%)							
Vertigo	19 (48.7)	11 (61.1)	18 (41.9)	19 (50.0)	37 (56.1)	8 (27.6)	0.130
Aural fullness	31 (79.5)	8 (44.4)	32 (74.4)	23 (60.5)	40 (60.6)	19 (65.5)	0.089
Tinnitus	33 (84.6)	16 (88.9)	38 (88.4)	34 (89.5)	54 (81.8)	22 (75.9)	0.624
Hypertension, No. (%)	4 (10.3)	5 (27.8)	4 (9.3)	5 (13.2)	10 (15.2)	5 (17.2)	0.496
Diabetes, No. (%)	5 (12.8)	3 (16.7)	2 (4.7)	4 (10.5)	6 (9.1)	0 (0.0)	0.290
Initial treatment time, mean, day	4.49 ± 1.82	4.72 ± 2.40	4.61 ± 2.01	4.82 ± 1.89	4.29 ± 2.13	5.35 ± 1.72	0.286
Adverse event, No. (%)							
Pain	18 (46.2)	10 (55.6)	17 (39.5)	18 (47.4)	20 (30.3)	13 (44.8)	0.310
Dizziness/vertigo	13 (33.3)	9 (50.0)	18 (41.9)	15 (39.5)	23 (34.8)	14 (48.3)	0.687
Ear infection	0 (0.0)	0 (0.0)	0 (0.0)	0 (0.0)	0 (0.0)	0 (0.0)	-
Tympanic membrane perforation	0 (0.0)	0 (0.0)	1 (2.3)	2 (5.3)	5 (7.6)	2 (6.9)	0.385
Recovery, No. (%)	15 (38.5)	7 (38.9)	34 (79.1)	24 (63.2)	44 (66.7)	19 (61.4)	0.002

*Quantitative data, such as age, initial treatment time and PTA was compared by a two-sample t-test or Wilcoxon signed-rank test. Categorical data, such as sex, tinnitus, aural fullness, vertigo, location of damaged ear, history of diabetes or hypertension, audiogram shapes and hearing recovery was compared by chi-squared test or Fisher's exact test*.

### Comparison of Side Events Among SSNHL Patients According to IT Injection Numbers

Side events among SSNHL patients according to IT injection numbers were compared in Table 4. Pain, dizziness/vertigo, ear infection, and tympanic membrane perforation could not differ significantly among these patients. No ear infection appeared in patients no matter how many IT injections. Although tympanic membrane perforation could not differ significantly, it seemed more likely to occur in patients with more IT injections.

## Discussion

In this study, we found that age, initial treatment time and audiogram shapes were associated with hearing recovery in SSNHL patients, which was consistent with previous studies. Moreover, by using logistic regression analysis, we found that the number of IT injections could be regarded as an independent factor that was positively associated with hearing recovery, and the optimal number of injections was 6.

The definitive pathological mechanism of SSNHL remains unclear. The possible pathogeneses of SSNHL have been postulated and include vascular, infectious, oxidative, immunomediated, and degenerative causes along with rupture of the cochlear membrane (19–22). SSNHL patients could recover their hearing even without treatment, with the proportion ranging from 32 to 70% (4, 23). But these numbers might be overestimated based on clinical practice (1). Hearing recovery relies on a variety of risk factors, including age, duration of hearing loss, accompanying symptoms, hearing characteristics and treatment (24). This study demonstrated that younger patients were more likely to recover hearing. This result was consistent with previous studies that indicated that advanced age was negatively correlated with rates of regaining preloss hearing thresholds (25). Audiogram shapes have been reported to correlate with hearing recovery in many studies (26, 27). Our study indicated similar results and showed that SSNHL patients with low-frequency hearing loss were 12.86 times more likely to recover hearing than patients with profound hearing loss. Vertigo can appear in SSNHL, and it is always regarded as a poor prognostic factor associated with hearing recovery because it always appears in profound hearing loss (28). This study suggested that SSNHL patients with vertigo were 0.49 times more likely to recover than those without vertigo. Our study also demonstrated that the effective group had a shorter duration from onset to treatment, which was similar to the previous literature (23, 24). However, in this study, PTA before treatment did not differ between the effective group and the ineffective group, which contrasts with many other studies. Previous studies have suggested that the initial PTA threshold was negatively correlated with hearing recovery (24, 29). Our study suggested that the evaluation of hearing recovery in SSNHL should depend on age, audiogram shapes, presence of vertigo, the number of IT injections and initial treatment time, rather than initial PTA.

Steroids are generally used for treating SSNHL due to their high effectiveness (1). They are always administered via systemic or IT routes. Recently, IT steroids have been increasingly widely used for SSNHL because they not only decrease the side effects caused by systemic steroids but also are more effective due to the high drug concentration in the cochlea (30). There is no consensus on IT injection frequency, and it varies from once daily to once weekly or less among studies (1, 7). Different frequencies of IT injections could cause different hearing outcomes. In Ermutlu et al.'s study, the number of IT injections was 3, and 84.2% of patients regained their hearing (31). Park used 6 injections of IT steroids over 2 weeks, and its effectiveness rate was 77.3% (32). Hong et al. reported that the hearing outcome of IT steroids injected once per day for 8 consecutive days was 78% {#29}. In fact, more frequent injection of IT steroids has a better effect due to the limited number of glucocorticoid receptors (GRs), which steroids must bind to have an effect. GRs are primarily distributed in the organ of Corti, followed by spiral ganglion neurons, the stria vascularis and the lateral cochlear wall (33, 34). Glucocorticoids bind to GRs to exert anti-inflammatory and immunosuppressive effects in SSNHL (35). In addition, more frequent IT injection could be expensive and cause many adverse effects, such as pain, transient dizziness, infection, and persistent tympanic membrane perforation (1). It is necessary to determine the optimal number of IT injections for reducing costs and side effects and having the best effect. In this study, we found that the number of IT injections was an independent factor associated with hearing recovery and that hearing recovery was positively correlated with the number of IT injections. According to the pharmacokinetics of IT dexamethasone in cochlea by Salt' study, IT dexamethasone could almost disappeared in both high-frequency and low-frequency cochlear regions after injected 24 h (36). IT dexamethasone was injected every day or every other day in this study. More frequent IT injections could result in higher dexamethasone does in cochlea. Theoretically, high cochlear concentrations could improve efficacy (37). After further analysis of the association between the number of IT injections and hearing recovery, we found that 6 injections was the optimal number of IT injections and resulted in 79.1% of patients regaining their hearing. This result was slightly higher than that of Park's study, in which 77.3% of patients effectively regained their hearing by means of 6 IT injections (32). This finding provides physicians with the important recommendation that 6 injections may be most appropriate for SSNHL patients treated with IT steroids.

There were some limitations in this study. First, this is a retrospective study including limited samples, which may cause selection bias and information bias. Second, the studied patients had only a 3-month follow-up hearing test. Some patients may have hearing recovery after 3 months. This limitation may underestimate the rates of hearing recovery. Third, patients in this study did not have hearing tests before SSNHL onset, which may result in the overdiagnosis of SSNHL. Fourth, if a patient did not recovery their hearing earlier, he would be willing to receive higher number of IT injections. This may cause selection bias and decrease the rate of hearing recovery in patients with higher number of IT injections.

In conclusion, the number of IT steroid injection was an independent factor that was positively associated with hearing recovery, and the optimal number of IT steroid injections was 6. Batroxobin plus higher number of IT steroid injections showed more effective for treating SSNHL.

## Data Availability Statement

The original contributions presented in the study are included in the article/supplementary material, further inquiries can be directed to the corresponding authors.

## Ethics Statement

The studies involving human participants were reviewed and approved by the Regional Ethical Standards Committee of the First Affiliated Hospital of Zhengzhou University. The patients/participants provided their written informed consent to participate in this study.

## Author Contributions

YW: built the models, analyzed the data, and drafted the manuscript. LW: collected the data. GG: collected the data and reviewed the manuscript. XM: reviewed the manuscript. LY: principal investigator and managed the study. FY: reviewed the manuscript. All authors contributed to the article and approved the submitted version.

## Funding

This study was supported by the National Natural Science Foundation of China (Grant Number: 82071034).

## Conflict of Interest

The authors declare that the research was conducted in the absence of any commercial or financial relationships that could be construed as a potential conflict of interest.

## Publisher's Note

All claims expressed in this article are solely those of the authors and do not necessarily represent those of their affiliated organizations, or those of the publisher, the editors and the reviewers. Any product that may be evaluated in this article, or claim that may be made by its manufacturer, is not guaranteed or endorsed by the publisher.
